# Delayed treatment with oleanolic acid attenuates tubulointerstitial fibrosis in chronic cyclosporine nephropathy through Nrf2/HO-1 signaling

**DOI:** 10.1186/1479-5876-12-50

**Published:** 2014-02-21

**Authors:** Yu Ah Hong, Ji Hee Lim, Min Young Kim, Eun Nim Kim, Eun Sil Koh, Seok Joon Shin, Bum Soon Choi, Cheol Whee Park, Yoon Sik Chang, Sungjin Chung

**Affiliations:** 1 Division of Nephrology, Korea University Guro Hospital, 148, Gurodong-ro, Guro-gu, Seoul 152-703, Republic of Korea; 2 Department of Internal Medicine, College of Medicine, The Catholic University of Korea, 222 Banpo-daero, Seoul 137-701, Republic of Korea; 3 Division of Nephrology, The Catholic University of Korea Yeouido St. Mary’s Hospital, 10, 63-ro, Yeongdeungpo-gu, Seoul 150-713, Republic of Korea; 4 Division of Nephrology, The Catholic University of Korea Incheon St. Mary’s Hospital, 56, Dongsu-ro, Bupyeong-gu, Incheon 403-720, Republic of Korea; 5 Division of Nephrology, The Catholic University of Korea Seoul St. Mary’s Hospital, 222, Banpo-daero, Seoul 137-701, Republic of Korea

**Keywords:** Cyclosporine, Kidney, Fibrosis, Oleanolic acid, Oxidative stress

## Abstract

**Background:**

Nuclear factor erythroid-2-related factor-2 (Nrf2) is known to protect against tissue injury by orchestrating antioxidant and detoxification responses to oxidative stress. This study investigated whether upregulation of Nrf2-dependent signaling by oleanolic acid (OA), which is known to activate Nrf2, could attenuate renal inflammation and fibrosis in cyclosporine (CsA)-induced kidney injury.

**Methods:**

Male ICR mice were divided into four treatment groups: Vehicle (VH, n = 6), VH + OA (n = 6), CsA (n = 8), and CsA + OA (n = 8). For the OA-treated groups, OA (25 mg/kg/day) was administered by intraperitoneal injection for the final week of the 4-week experimental period. Renal function, morphologies and signaling were evaluated at the end of the study.

**Results:**

Treatment with CsA resulted in decreased kidney function and urine osmolality and increased urine volume and urinary albumin levels. The CsA-induced changes were improved by OA treatment. Specifically, administration of OA decreased tubulointerstitial fibrosis and inflammation scores that were increased in CsA-treated mice. Furthermore, OA treatment decreased urinary 8-hydroxy-2′-deoxyguanosine (8-OHdG) and 8-epi-prostaglandin F2α (8-iso-PGF2α) levels. The beneficial effects of OA were attributed to an increased ratio of nuclear/total Nrf2 and subsequently enhanced expression of heme oxygenase (HO)-1, as well as a stable level of Kelch-like ECH-associated protein 1 (Keap1) expression, indicating that OA enhanced nuclear translocation of Nrf2. Increased apoptotic cell death and a high ratio of B cell leukaemia/lymphoma 2 (Bcl-2)-associated X protein (Bax) to Bcl-2 in CsA-treated mice were also significantly ameliorated by OA treatment.

**Conclusion:**

Our results suggest that OA activates Nrf2/HO-1 signaling in chronic CsA nephropathy, which may have beneficial effects on inflammation and oxidative stress.

## Background

Cyclosporine A (CsA) is one of the most effective and widely used immunosuppressants in solid organ transplantation and autoimmune disease. Despite the beneficial effects of CsA, its use in the clinic is limited by its nephrotoxic potential. CsA-induced nephropathy is characterized by progressive renal insufficiency, arteriopathy of the afferent arterioles, tubulointerstitial inflammation, and striped fibrosis [1]. Although the exact mechanism of CsA-induced nephrotoxicity remains obscure, oxidative stress and apoptotic cell death can play a pivotal role in producing structural and functional kidney impairment in CsA-induced renal injury [2,3]. Therefore, it is necessary to control inflammation, apoptosis, and fibrosis associated with oxidative stress in order to delay progression of chronic CsA nephropathy.

Studies on the renal protective response and pathways responsible for activation of intracellular specific signaling molecules or genes should aid in our understanding of oxidative stress and development of future therapeutics. Recently, nuclear factor E2-related factor 2 (Nrf2) was found to be a critical transcription factor that binds to antioxidant response elements (AREs) present in the promoter region of a number of genes encoding antioxidant and phase 2 enzymes, including glutathione peroxidase, catalase, and superoxide dismutase (SOD) [4,5]. Nrf2 appears to function upon being released from its repressor Kelch-like ECH-associated protein 1 (Keap1) by sensing cytoplasmic oxidative stress or other specific chemical agents [6,7]. Heme oxygenase (HO)-1, an important Nrf2 target gene, catalyzes heme metabolism, yielding iron, carbon monoxide and bilirubin. Likewise, HO-1 is recognized as a protective gene in the kidney involved in degradation of pro-oxidant heme, resulting in production of anti-inflammatory, antioxidant, and anti-apoptotic metabolites [8].

The cytoprotective effects of Nrf2 are supported by previous studies showing that Nrf2 gene ablation intensifies inflammation, oxidative stress and histological changes [9,10]. Oleanolic acid (OA), a known activator of Nrf2, is a natural pentacyclic triterpenoid that has hepatoprotective, anti-inflammatory, antioxidant, and anticancer activities [11]. Recently, OA has been reported to have renoprotective effects in experimental animals using toxicants and streptozocin [12,13]. However, there have been no studies on the renoprotective effects of OA in a mouse model of chronic CsA nephropathy. Thus, the aim of this study was to investigate whether upregulation of Nrf2-depedent antioxidative signaling by OA could attenuate renal oxidative stress and fibrosis in CsA-induced nephropathy. In addition, we delayed administration of OA to determine whether Nrf2 activated by OA treatment confers renoprotective effects when initiated after the onset of CsA-induced damage.

## Materials and methods

### Experimental design

Five-week-old male ICR mice (DBL, Chungcheongbuk-do, Republic of Korea), initially weighing 15 to 20 g, were housed in a temperature- and light-controlled environment and allowed free access to a low-salt diet (0.01% sodium; Research Diets, NJ, USA) and tap water. Mice were randomized into four subgroups and treated daily for 28 days: (1) Vehicle-treated control group (VH, n = 6) was given a daily subcutaneous injection of vehicle (olive oil 1 mL/kg, Sigma-Aldrich, St. Louis, MO, USA); (2) Vehicle plus OA-treated group (VH + OA, n = 6) was given a daily subcutaneous injection of olive oil (1 mL/kg), and in this group, OA (25 mg/kg; Sigma-Aldrich, St. Louis, MO, USA) was injected intraperitoneally daily for one week; (3) CsA only-treated group (CsA, n = 8) was given a daily subcutaneous injection of CsA (30 mg/kg; Chong Kun Dang, Seoul, Republic of Korea); and (4), the CsA plus OA-treated group (CsA + OA, n = 8) was given a daily subcutaneous injection of CsA, and OA was given by daily intraperitoneal injection for the final week of the experimental period. The doses of CsA [14,15] and OA [16] were selected based on previous reports. The experimental protocol was approved by the Institutional Animal Care and Use Committee of The Catholic University of Korea (CUMC-2012-0093-02).

### Measurement of basic parameters

Before sacrifice, animals were housed individually in metabolic cages (Nalge, Rochester, NY, USA) for 24-hr urine collection. Albuminuria and urine creatinine were measured using ELISA kits (Exocell, Philadelphia, PA, USA). Measurement of serum creatinine concentrations and urine osmolality was performed at Samkwang Medical Laboratories (Seoul, Korea) using enzymatic colorimetric methods (Modular DPP system, Roche, Hamburg, Germany). Creatinine clearance was calculated using a standard formula (urine creatinine (mg/dL) × urine volume (mL/24 h)/serum creatinine (mg/dL) × 1440 (min/24 h)). Whole-blood CsA concentrations were quantified using liquid chromatography-tandem mass spectrometry with an API3200 LC-MS System (Applied Biosystems/MDS Sciex, Foster City, CA, USA) equipped with an electrospray ionization interface to generate negative ions [M + NH4]^+^.

### Histopathology

On the day of sacrifice, kidneys were retrieved, washed with heparinized saline, fixed in a periodate-lysine-paraformaldehyde solution, and embedded in wax. After dewaxing, 4 μm sections were processed and stained with hematoxylin-eosin (H&E), periodic acid Schiff (PAS), and Masson’s trichrome. Tubulointerstitial fibrosis (TIF) was defined as a matrix-rich expansion of the interstitium with tubular dilatation, atrophy, cast formation, and sloughing of tubular epithelial cells or thickening of the tubular basement membrane. At least 20 fields per section were assessed by counting the percentage of injured areas per field of cortex at 200× magnification with a color-image analyzer (TDI Scope Eye Version 3.5 for Windows; Olympus, Japan).

### *In situ* TUNEL assay

Apoptosis was assessed using terminal deoxynucleotidyl transferase-mediated biotin nick end-labeling (TUNEL) assay. Detection of apoptotic cells in formalin-fixed, paraffin-embedded tissue was performed by *in situ* TUNEL using the ApopTag *In Situ* Apoptosis Detection Kit (Chemicon-Millipore, Billerica, MA, USA) according to the manufacturer’s protocol. TUNEL positive cells were evaluated at 400× magnification.

### Assessment of markers for renal oxidative stress

Twenty-four-hour urinary concentrations of 8-hydroxy-2′-deoxyguanosine (8-OHdG; OXIS Health Products, OR, USA) and 8-epi-prostaglandin F2α (8-epi-PGF2α; OxisResearch, CA, USA) were measured using a competitive enzyme-linked immunosorbent assay according to the manufacturer’s protocol. Lipid peroxidation as an index of oxidative stress was determined by assaying malondialdheyde (MDA) production with the thiobarbituric Acid Reactive Substance (TBARS) test (OxiSelect MDA Adduct ELISA Kit, Cell Biolabs Inc., CA, USA).

### Immunohistochemistry for cleaved caspase-3 and α-SMA

Presence of cleaved caspase-3 and α-smooth muscle actin (α-SMA) was determined by immunohistochemistry. Briefly, small blocks of kidney were immediately fixed in 10% buffered formalin for 24 h before being embedded in paraffin. Next, 4 μM thick sections of renal tissues were incubated overnight with anti-cleaved caspase-3 (1:100; Abcam, Cambridge, UK) or anti-α-SMA (1:500; Abcam, Cambridge, UK) in a humidified chamber at 4°C. The primary antibodies were localized with a peroxidase-conjugated secondary antibody and developed using the Vector Impress kit (Vector Laboratories, Burlingame, CA, USA) and 3, 3-diamninobenzidine substrate solution with nickel chloride enhancement. Sections were then dehydrated in ethanol, cleared in xylene, and mounted without counterstaining. All of these sections were examined in a blinded manner using light microscopy (Olympus BX-50, Olympus Optical, Tokyo, Japan). For the quantification of the proportional areas of staining, approximately 20 views (400× magnifications) were randomly located in the renal cortex and the corticomedullary junction of each slide.

### Western blotting

For Western blot analysis, total protein of renal cortical tissues was extracted with a Pro-Prep Protein Extraction Solution (Intron Biotechnology, Gyeonggi-do, Korea) according to the manufacturer’s instructions. Nuclear extracts, which were used for Nrf2 immunoblotting, were prepared with the NE-PER nuclear kit as previously described (Pierce Biotechnology, Rockford, IL) [17]. Western blotting was performed for nuclear Nrf2, total Nrf2, Keap1, HO-1, NAD(P)H quinone oxidoreductase-1 (NQO1), B cell leukaemia/lymphoma 2 (Bcl-2), Bcl-2-associated X protein (Bax), SOD1, SOD2, and catalase. Specifically, proteins were separated by SDS-PAGE, transferred to nitrocellulose membranes, and detected with the following antibody concentrations: Nrf2 (1:1000; Santa Cruz Biotechnology Inc, Texas, USA), Keap1 (1:1000; Santa Cruz Biotechnology Inc, Texas, USA), HO-1 (1:1000; BD Biosciences, California, USA), NQO1 (1:1000; Santa Cruz Biotechnology Inc, Texas, USA), Bcl-2 (1:500; Santa Cruz Biotechnology Inc, Texas, USA), Bax (1:500; Santa Cruz Biotechnology Inc, Texas, USA), SOD1 (1:5000; Assay Designs, MI, USA), SOD2 (1:10000; Abcam, Cambridge, UK), Catalase (1:2000; Abcam, Cambridge, UK), and β-actin (1:10000; Sigma-Aldrich, MO, USA).

### Statistical analysis

Data are expressed as the mean ± SEM. Multiple comparisons between groups were performed by one-way analysis of variance with post hoc tests. Statistical significance was assumed as p < 0.05.

## Results

### Renal functional parameters

Table 1 shows the changes in functional parameters among all groups at the end of the 4-week study period. Compared with VH and VH + OA, the CsA-treated groups CsA and CsA + OA had a slightly reduced body weight, although the difference was not statistically significant. Urine volume and 24-hour albuminuria were significantly increased in the CsA group compared with the VH and VH + OA groups. OA treatment improved albuminuria and decreased urine volume to the level of the control groups VH and VH + OA. There was a significant decrease in urine osmolality in CsA mice compared with those in the VH, and a significant increase in CsA + OA group. We also observed decreased creatinine clearance in mice given CsA compared to mice in the VH group. However, administration of OA inhibited the decline in creatinine clearance in CsA-treated mice.

**Table 1:** Biochemical and physical characteristics of the four groups at the end of the 4-week period

	**VH (n = 6)**	**VH + OA (n = 6)**	**CsA (n = 6)**	**CsA + OA (n = 8)**
Body weight (g)	35.77 ± 5.25	34.3 ± 1.45	33.97 ± 2.34	32.78 ± 4.46
Kidney weight (g)	0.23 ± 0.04	0.23 ± 0.02	0.18 ± 0.04	0.22 ± 0.03
Urine volume (mL)	1.53 ± 0.97	2.53 ± 1.71	7.15 ± 5.15^b^	1.4 ± 1.96
Serum Cr (mg/dl)	0.21 ± 0.02	0.26 ± 0.02	0.30 ± 0.11	0.25 ± 0.06
Cr Clearance (mL/min/100 g BW)	0.72 ± 0.15	0.44 ± 0.09	0.26 ± 0.16^a^	0.52 ± 0.19
24 hr albuminuria (μg/day)	23.90 ± 4.48	26.75 ± 11.88	150.99 ± 57.42^a^	57.34 ± 7.44
Urine osmolality (mOsm/kg)	2088.67 ± 963.44	810.67 ± 181.88	582.00 ± 208.89^b^	2143.33 ± 547.01
CsA concentration (ng/mL)	N/A	N/A	1792.34 ± 63.08	1764.40 ± 28.56

Data are means ± SEM. ^a^p < 0.05 compared with vehicle, ^b^p < 0.05 compared with other groups. VH = vehicle-treated group; VH + OA = Vehicle plus oleanolic acid-treated group; CsA = cyclosporine-treated group; CsA + OA = cyclosporine plus oleanolic acid-treated group; Cr = creatinine; BW = body weight; N/A = not available.

### Effects of oleanolic acid on renal morphological changes

**Figure 1:**
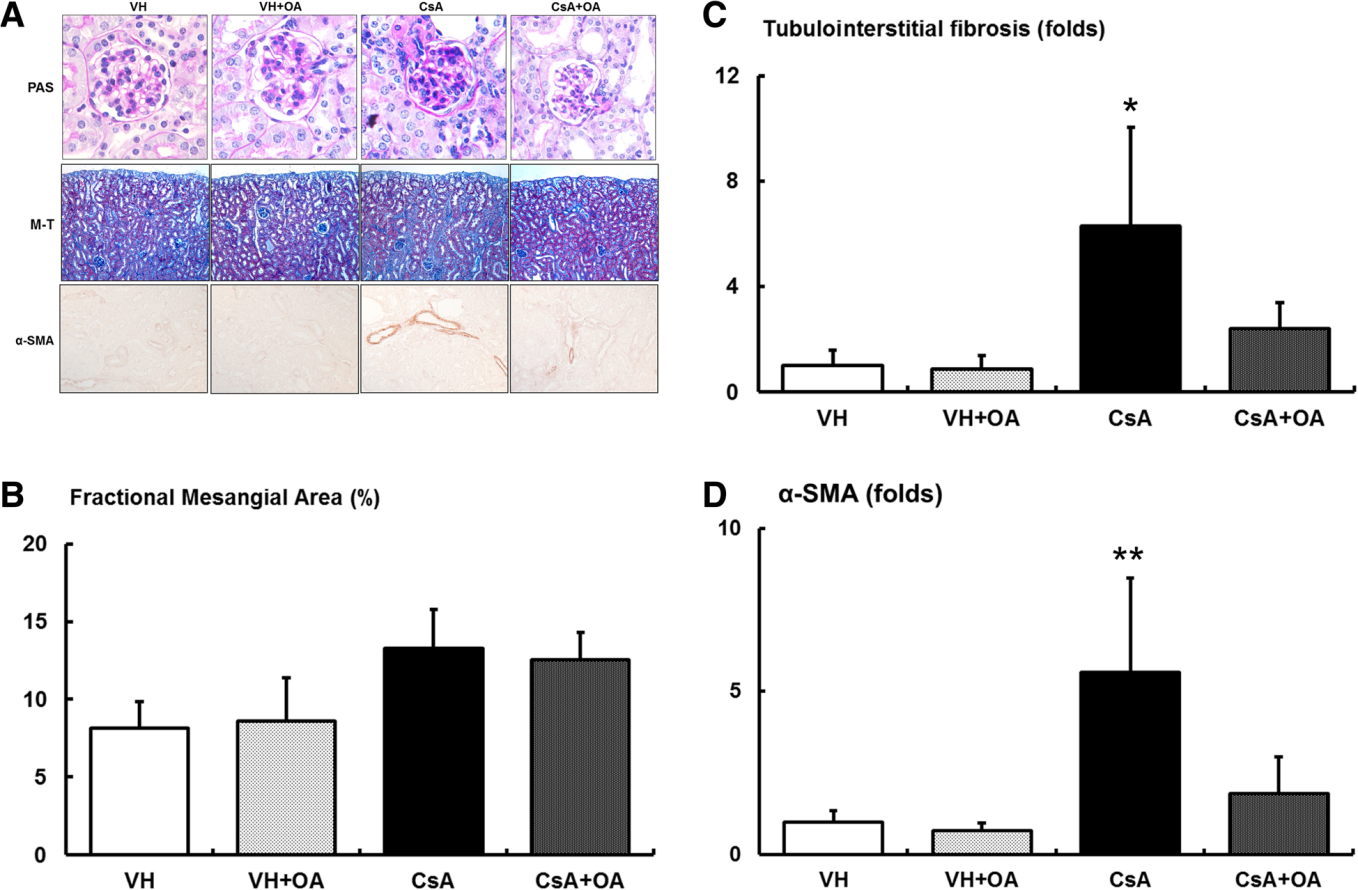
**Effects of oleanolic acid (OA) on renal morphological changes. (A)** Representative renal sections stained with periodic acid-Schiff (PAS; original magnifications, ×400), Masson-trichrome (M-T; original magnifications, ×400), and α-smooth muscle actin (α-SMA; original magnifications, ×200). **(B)** Quantitative analyses of the results for the mesangial fractional area (%). **(C)** Quantitative analyses of the results for the tubulointerstitial fibrosis area. *p <0.01 vs. other groups. **(D)** Quantitative analyses of the results for α-SMA. **p <0.05 vs. other groups. VH = vehicle-treated group; VH + OA = vehicle plus oleanolic acid-treated group; CsA = cyclosporine-treated group; CsA + OA = cyclosporine plus oleanolic acid-treated group.

There was no significant difference of fractional mesangial area among all study groups (Figure 1A and 1B). TIF was produced in the cortex of the kidney of CsA-treated mice. Quantitative analysis showed that renal fibrosis was significantly increased in mice given CsA compared with VH and VH + OA mice. By contrast, OA treatment significantly decreased TIF in CsA-treated mice (Figure 1A and 1C). Administration of OA significantly inhibited the CsA-induced increase in α-SMA expression (Figure 1A and 1D).

### Effects of oleanolic acid on renal expression of Nrf-2, Keap1, and the antioxidant defense system

**Figure 2:**
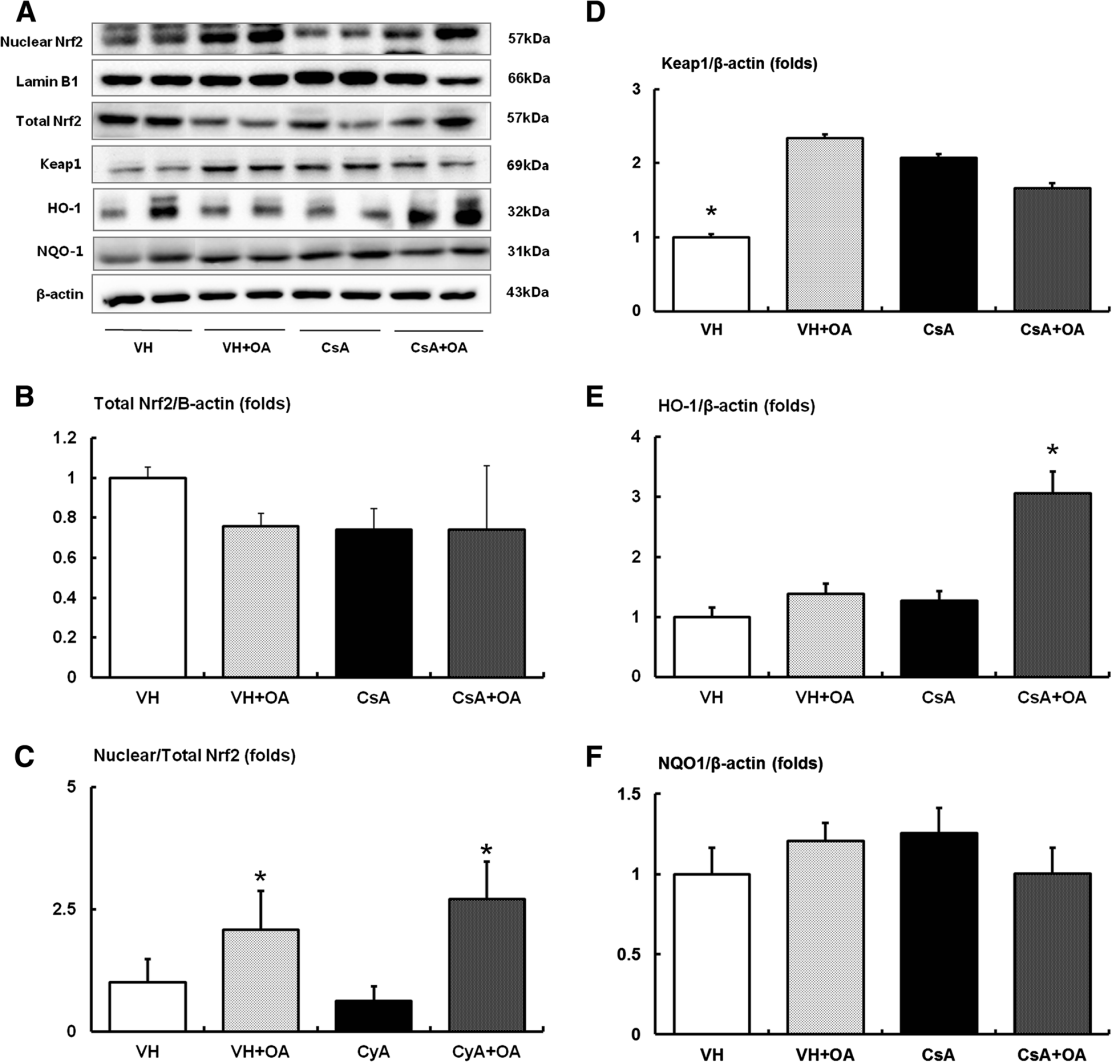
**Effects of oleanolic acid (OA) on nuclear/total Nrf2, Keap1, HO-1 and NQO1 expressions in chronic CsA nephropathy. (A)** Representative Western blot showing the effects of OA on nuclear/total Nrf2, Keap1, HO-1 and NQO1 expression in chronic CsA nephropathy. **(B)** Quantitative analyses for total Nrf2/β-actin. There were no significant differences identified by quantitative analysis for immunoblotting of total Nrf2 among the experimental groups. **(C)** Quantitative analyses for nuclear/total Nrf2. There was increased nuclear/total Nrf2 in the CsA + OA compared with CsA. *p <0.05 vs. VH and CsA groups. **(D)** Quantitative analyses for Keap1/β-actin. *p <0.05 vs. other groups. **(E)** Quantitative analyses for HO-1/β-actin. Quantification of HO-1 immunoblotting revealed a significant increase in CsA + OA mice compared with other groups. *p <0.05 vs. other groups. **(F)** Quantitative analyses for NQO1/β-actin. There were no significant differences in the expression of NQO1 among the experimental groups. Nrf2 = nuclear factor erythroid-2-related factor-2; Keap1 = Kelch-like ECH-associated protein 1; HO-1 = heme oxygenase-1; NQO1 = NAD(P)H quinone oxidoreductase-1; VH = vehicle-treated group; VH + OA = vehicle plus oleanolic acid-treated group; CsA = cyclosporine-treated group; CsA + OA = cyclosporine plus oleanolic acid-treated group.

We next evaluated the effect of OA on the Nrf2/Keap1 signaling pathway (Figure 2A). The expressions of total Nrf2 in the kidney were mildly decreased in VH + OA, CsA and CsA + OA groups, but there was no significant difference between those with or without OA treatment (Figure 2B). On closer inspection, administration with OA markedly increased the level of nuclear Nrf2 in the kidneys of mice. Consequently, the intra-renal nuclear Nrf2/total Nrf2 ratio was significantly increased in the CsA + OA group compared with the CsA group (Figure 2C). The expression of the Nrf2 repressor Keap1 significantly increased in the VH + OA, CsA and CsA + OA groups compared with VH group (Figure 2D). Next, we measured the expression of two Nrf2 target proteins, NQO1 and HO-1. Administration of OA in CsA-treated mice resulted in a significant increase in HO-1 expression compared with the VH and CsA groups (Figure 2E). Conversely, there was no statistically significant difference in NQO1 levels among any of the groups (Figure 2F).

**Figure 3:**
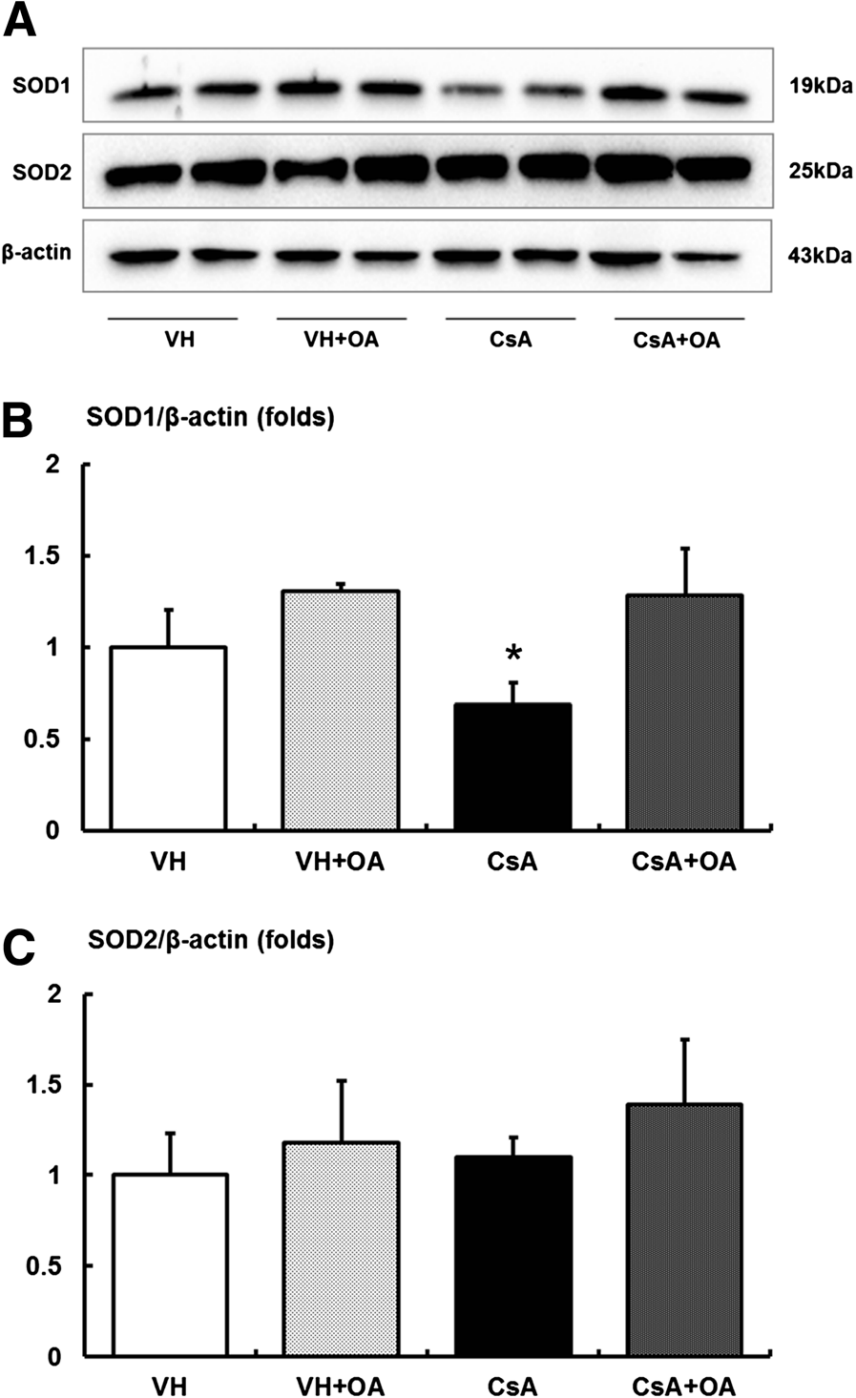
**Effect of oleanolic acid (OA) on expressions of SODs in chronic CsA nephropathy. (A)** Immunoblotting for the target molecules of SOD1 and SOD2 showing the effects of OA in chronic CsA nephropathy. **(B)** Quantitative analyses for SOD1/β-actin. The expression of SOD1 was significantly decreased in CsA but increased in CsA + OA mice. *p <0.05 vs. other groups. **(C)** Quantitative analyses for SOD2/β-actin. There were no significant differences in the expression of SOD2 among the experimental groups. SOD = superoxide dismutase; VH = vehicle-treated group; VH + OA = vehicle plus oleanolic acid-treated group; CsA = cyclosporine-treated group; CsA + OA = cyclosporine plus oleanolic acid-treated group.

**Figure 4:**
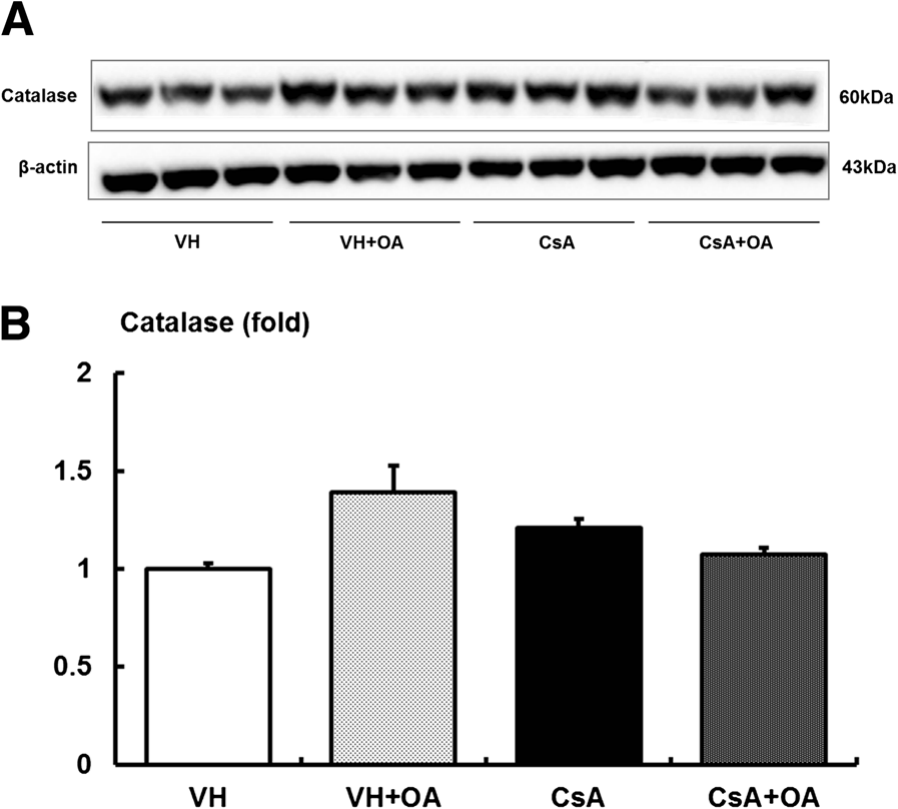
**Effect of oleanolic acid (OA) on catalase expression in chronic CsA nephropathy. (A)** Immunoblotting for the target molecule catalase in chronic CsA nephropathy. **(B)** Quantitative analyses for catalase/β-actin. There were no significant differences in the expression of catalase among the experimental groups. VH = vehicle-treated group; VH + OA = vehicle plus oleanolic acid-treated group; CsA = cyclosporine-treated group; CsA + OA = cyclosporine plus oleanolic acid-treated group.

The protein levels of SOD1 and SOD2 were also evaluated (Figure 3A). SOD1 levels were significantly lower in the CsA group compared with VH and VH + OA groups. In CsA-treated mice, administration of OA restored levels of SOD1 to those found in the VH and VH + OA groups (Figure 3B). However, the expression of SOD2 was not different among groups (Figure 3C). Similarly, OA had no effect on catalase levels (Figure 4).

**Figure 5:**
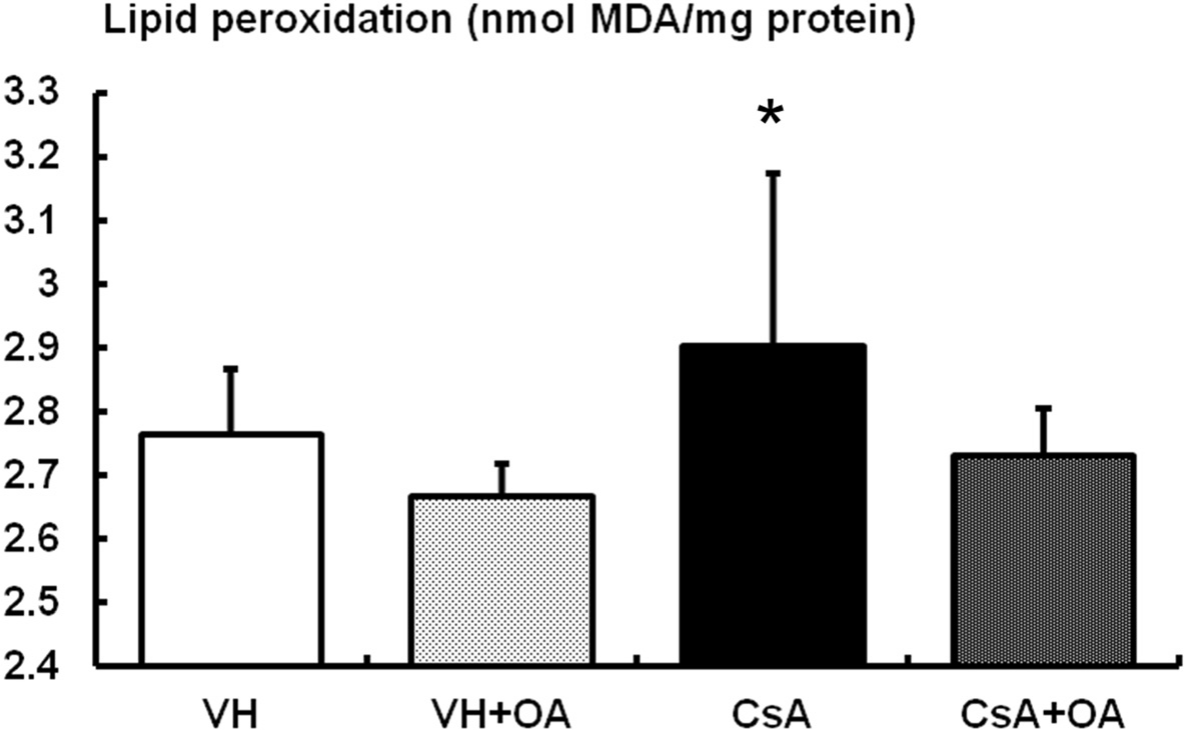
**Assay for concentrations of the lipid peroxidation marker malonedialdehyde (MDA) in renal cortical tissue among the experimental groups.** *p <0.05 vs. other groups. VH = vehicle-treated group; VH + OA = vehicle plus oleanolic acid-treated group; CsA = cyclosporine-treated group; CsA + OA = cyclosporine plus oleanolic acid-treated group.

**Figure 6:**
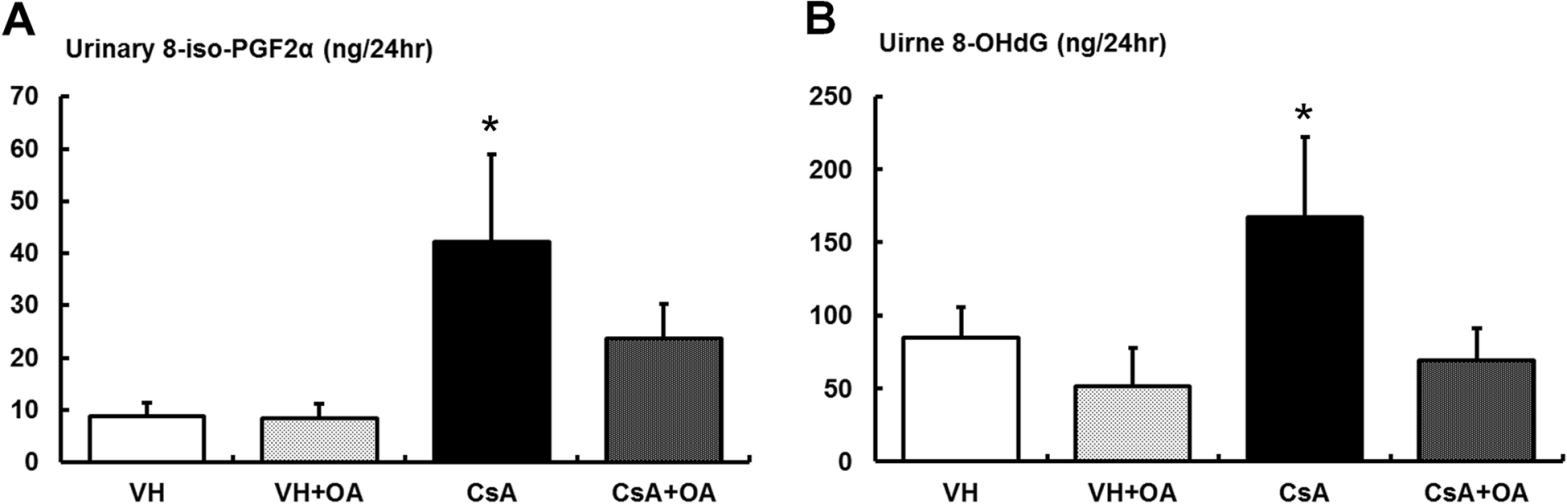
**Assessment of urinary markers for CsA-induced oxidative stress. (A)** 24 hr urinary 8-iso-PGF2α concentrations from the experimental groups is shown. *p <0.05 vs. other groups. **(B)** 24 hr urinary excretion of 8-OHdG from the experimental groups. *p <0.05 vs. other groups. 8-iso-PGF2α = 8-epi-prostaglandin F2α; 8-OHdG = 8-hydroxy-2′-deoxyguanosine; VH = vehicle-treated group; VH + OA = vehicle plus oleanolic acid-treated group; CsA = cyclosporine-treated group; CsA + OA = cyclosporine plus oleanolic acid-treated group.

We also observed increased levels of MDA, a stable indicator of oxidative stress, in CsA-treated mice; however, administration of OA reversed the elevation of MDA (Figure 5). In addition, the levels of 24-hour urinary 8-iso-PGF2α and 8-OHdG were higher in the CsA compared with control groups, and were attenuated by treatment with OA (Figure 6).

### Effects of oleanolic acid on renal apoptosis

**Figure 7:**
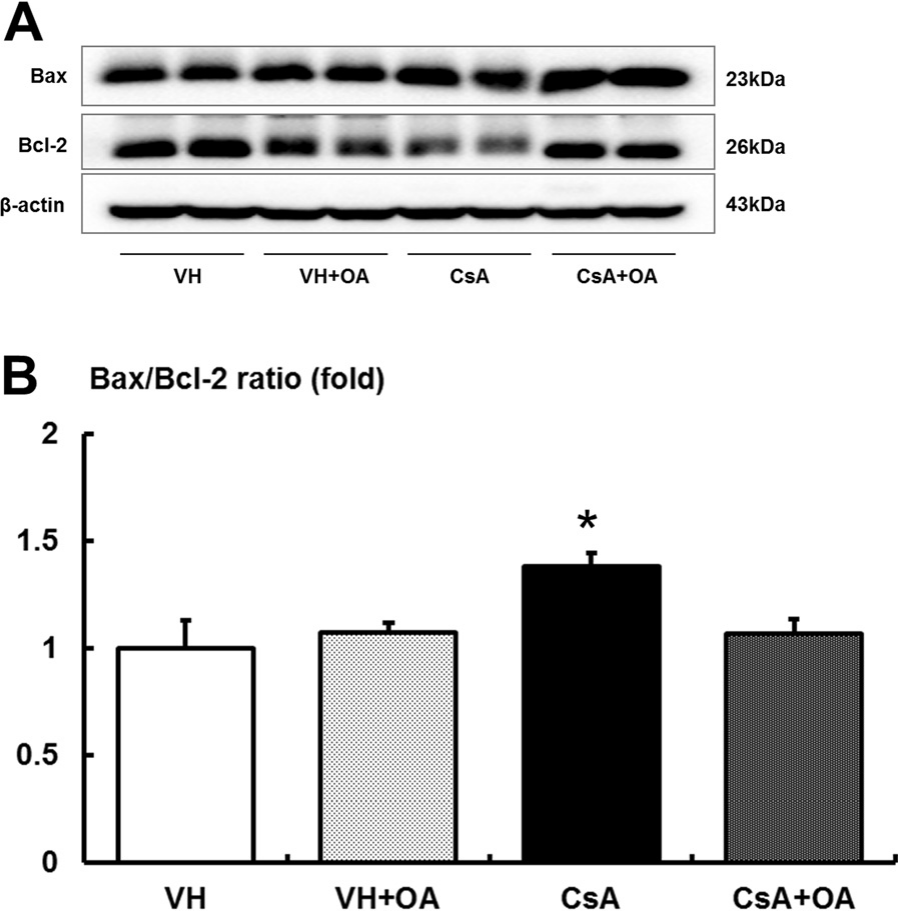
**Effect of oleanolic acid (OA) on Bax and Bcl-2 expressions in chronic CsA nephropathy. (A)** Representative Western blot analysis of Bax and Bcl-2 levels. **(B)** Quantitative analyses of the results of the Bax/Bcl-2 ratio. The Bax/Bcl-2 ratio was significantly reduced in the CsA + OA group compared with the CsA group. *p <0.05 vs. other groups. VH = vehicle-treated group; VH + OA = vehicle plus oleanolic acid-treated group; CsA = cyclosporine-treated group; CsA + OA = cyclosporine plus oleanolic acid-treated group.

**Figure 8:**
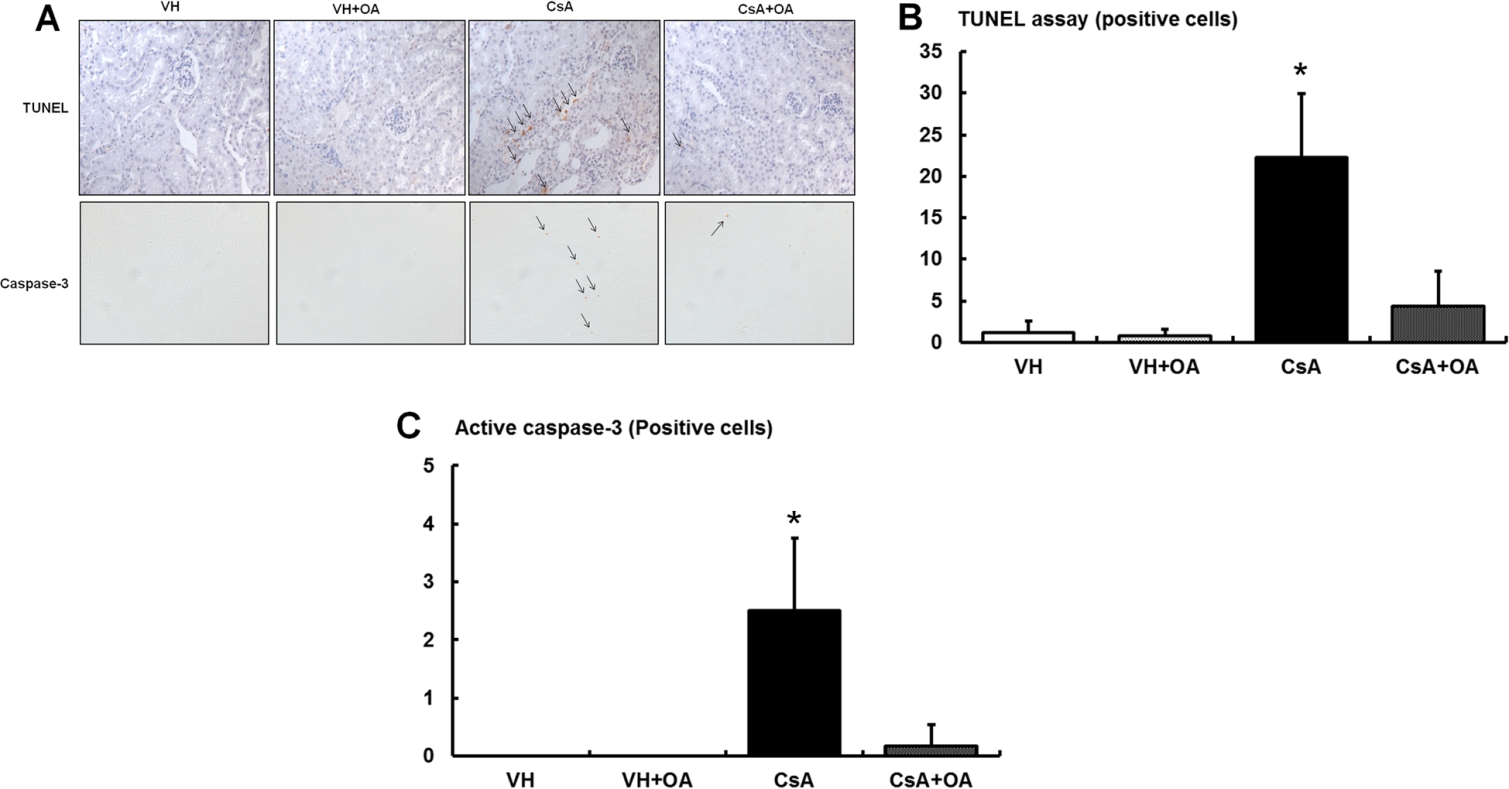
**Effect of oleanolic acid (OA) on apoptosis in chronic CsA nephropathy. (A)** TUNEL assay and immunohistochemical stain of active caspase-3 in kidney tissues of the experimental groups. **(B)** Quantitative analysis of TUNEL-positive nuclei in the experimental groups. The number of TUNEL-positive nuclei was significantly reduced in the CsA + OA group compared with the CsA group. *p <0.01 vs. other groups. **(C)** Quantitative analysis of the cleaved caspase-3 results. Cleaved caspase-3, a pro-apoptotic marker, was significantly decreased in CsA + OA compared with CsA. *p <0.01 vs. other groups. TUNEL = terminal deoxynucleotidyl transferase-mediated biotin nick end-labeling; VH = vehicle-treated group; VH + OA = Vehicle plus oleanolic acid-treated group; CsA = cyclosporine-treated group; CsA + OA = cyclosporine plus oleanolic acid-treated group.

CsA treatment suppressed the expression of anti-apoptotic marker Bcl-2 and increased the expression of pro-apoptotic markers Bax and cleaved caspase-3 (Figures 7 and 8). There was a significant increase in the Bax/Bcl-2 protein ratio for the CsA group compared with the VH and VH + OA groups. However, the elevated Bax/Bcl-2 ratio was significantly attenuated in the CsA + OA group (Figure 7). Furthermore, the number of TUNEL-positive cells and the expression of cleaved caspase-3 were significantly higher in the CsA group compared with the VH and VH + OA groups (Figure 8A). However, administration of OA to CsA-treated mice significantly reduced the number of TUNEL-positive cells by approximately 80% compared with the CsA group without OA (Figure 8B). In addition, OA attenuated the activation of cleaved caspase-3 (Figure 8C).

## Discussion

The current study demonstrated that OA treatment ameliorated both renal dysfunction and histopathology in chronic CsA-induced nephropathy. The renoprotective mechanism of OA towards CsA-induced renal injury appeared to be associated with improvement of interstitial inflammation, fibrosis, and apoptotic cell death, as well as attenuation of oxidant stress through association with enhanced nuclear translocation of Nrf2 and subsequent activation of downstream antioxidant enzyme targets.

Numerous studies have demonstrated the relationship between pathogenesis and progression of renal diseases and oxidative stress [18-21]. CsA treatment is known to induce oxidative stress injury by increasing the production of reactive oxygen species (ROS) and MDA and decreasing the activities of antioxidant enzymes such as SOD and glutathione peroxidase [22,23]. Considering the findings that impairment of Nrf2 activity and consequent down-regulation of its antioxidant and detoxifying target genes products play a major role in the pathogenesis and progression of kidney disease [24,25], pharmacological activation of Nrf2 may be useful for treating CsA-induced renal injuries.

OA is widely distributed in the plant kingdom as a free acid or an aglycone of tripenoid saponins [26]. OA can be easily obtained in high yield from olive pulp remaining after the oil is pressed from the olive fruit, as well as from olive leaves that are usually discarded after trees are pruned [27]. Previous studies have shown that OA has potent antioxidant activity and may protect several types of cells and tissues against oxidative stress [28]. The antioxidant effects of OA appear to be mediated to a great extent through activation of Nrf2 [16,17]. Nrf2 is a basic leucine zipper transcription factor that regulates many genes encoding detoxifying and antioxidant enzymes, including NQO1, GSH S-transferase, HO-1, glutamate cysteine ligase, peroxiredoxin, and GSH peroxidase, all of which contribute to cellular protection by removing ROS including superoxide anions, hydrogen peroxide, and hydroxyl radicals [29]. The inactive form of Nrf2 is localized in the cytoplasm bound to Keap1, a cytoskeleton-associated protein. Nrf2 activation appears to occur by various mechanisms including release of Nrf2 from Keap1, downregulation of Keap1 expression, disruption of the Keap1-Cullin-3 complex, and nuclear translocation of Nrf2 [30]. Upon exposure to oxidative stress, Nrf2 dissociates from Keap1 and translocates into the nucleus where it dimerizes with small Maf binding protein [31]. In our study, we found that administration of OA in CsA-treated mice did not result in an increase in Keap1 expression, but did increase the ratio of nuclear/total Nrf2 in CsA-treated kidneys. Although Nrf2 activation is an initial cellular adaptive response that occurs as soon as cells are challenged by oxidative stress [31], prolonged oxidative stress and inflammation appears to disturb nuclear translocation of Nrf2 in chronic CsA nephropathy. Based on the findings of our study, OA may be considered as a facilitator of nuclear translocation of cytoplasmic Nrf2 released from Keap1 following oxidative stress resulting from CsA treatment.

After translocating to the nucleus, Nrf2 is involved with protection of the cell against oxidative stress through ARE-mediated induction of several phase 2 detoxifying and antioxidant enzymes such as HO-1 [32,33]. HO-1 is expressed at low levels within normal kidneys and is induced in response to tubulointerstitial injury. In proteinuric human kidney disease, HO-1 protein is induced in tubular epithelial cells, more prominently in distal tubules than proximal tubules, but is not expressed in resident glomerular cells. However, expression of HO-1 protein in proximal tubules, but not in distal tubules, correlates with proteinuria, hematuria, and tubulointerstitial disease [34,35]. A previous study suggested that downregulation of HO-1 by CsA may be an underlying mechanism of CsA-induced nephrotoxicity [31]. The propensity for upregulation of HO-1 protein to occur in renal tubules but not in glomerular cells in kidney disease may be related to the differential sensitivity and response to oxidant stress exhibited by these cells. Consistent with the results of previous studies, our results showed that administration of OA in chronic CsA nephropathy shifted Nrf2 localization from the cytoplasm to the nucleus in kidneys and subsequently activated HO-1 expression compared with the CsA only-treated group. Unlike previous studies [17,28,36], we did not observe an increase in renal expressions of NQO1, SOD2 and catalase induced by OA in our model of CsA nephropathy. It has been reported that the total cellular balance of pro-oxidants and anti-oxidant intermediates determines how signaling mechanisms respond to various insults [37]. Therefore, the degree of oxidative stress or the innate antioxidant system’s response to insults may determine the detailed induction of specific antioxidant genes regulated by Nrf2.

Apoptosis is an essential process in the development and tissue homeostasis of most multicellular organisms, and deregulation of apoptosis has been implicated in the pathogenesis of CsA-induced nephropathy [38,39]. CsA induces Bax aggregation and translocation to the mitochondria causing activation of caspase-9, which in turn cleaves and activates caspase-3, an effector caspase, resulting in loss of the mitochondrial transmembrane potential and apoptotic cell death [38]. In the present study, OA administration appeared to protect renal interstitial cells against apoptosis induced by CsA. Notably, the Bax/Bcl-2 ratio, which corresponds with cell survival in response to apoptotic stimuli, returned to normal levels compared with the control group after administration of OA in CsA-treated mice.

There are some apparent discrepancies in the literature regarding whether the synthetic pentacyclic oleanane derived from OA has anti-apoptotic or pro-apoptotic effects. In an experiment using mouse hepatoma and human hepatopblastoma cells, Nrf2 was shown to control cell apoptosis via upregulation of Bcl-2 transcription and downregulation of Bax [40]. On the contrary, synthetic pentacyclic oleananes have been reported to induce apoptosis in vitro in human and rodent cancer cells including cells derived from myelomas, leukemia, and sarcomas [27]. These differences can be better understood by considering the specific triterpenoids and cell types studied [27]. Depending on the circumstances, Nrf2 activated by OA appears to be involved in regulating the balance between anti-apoptotic and pro-apoptotic signaling rather than centralizing the direction of a sole signaling pathway.

There were several interesting aspects of this study in addition to those described above. Although administration of OA in normal control mice increased the nuclear/total Nrf2 ratio as well as elevated the level of Keap1, the levels of antioxidant enzymes were not significantly altered. This observation suggested that other factors must function in parallel to mediate proper regulation of the downstream targets of Nrf2. In addition, the optimal dose and treatment period of OA for preventing CsA-induced injury has not yet been determined. In our preliminary studies, several additional experiments were performed. First, mice were treated with CsA in the absence or presence of OA for four weeks in order to evaluate its potential as an early or preventative treatment. However, the early treatment schedule was suspended two weeks after starting administration of OA because of significant weight loss and high mortality in mice treated with both CsA and OA. Furthermore, a high dose of OA (50 mg/kg/day) also resulted in significant weight loss and decreased activity in mice 4–5 days after starting administration of OA. Together, these findings suggested that mice do not tolerate high-dose or long-term treatment of OA in a model of CsA-induced nephropathy.

## Conclusions

In conclusion, the results of the present study demonstrated that delayed administration of OA could provide renoprotective effects against CsA-induced renal injury. The antioxidant potential of OA might be directly correlated with the increased expression of nuclear Nrf2 and subsequently HO-1, which in turn may have surmounted the oxidative stress generated by CsA. In this way, Nrf2 activated by OA may be an important therapeutic target for controlling CsA-induced nephrotoxicity.

## Competing interests

The authors declare that they have no competing interests.

## Authors’ contributions

The experiment was designed and implemented by YAH, SJS and SC. Data were analyzed by YAH, JHL, MYK, ENK and ESK. YAH, BSC, CWP and YSC participated in data acquisition and interpretation. YAH and SC prepared the manuscript. CWP and SC supervised overall project. All authors read and approved the final version of manuscript.
